# Targeted Sequencing of Human Satellite 2 Repeat Sequences in Plasma cfDNA Reveals Potential Breast Cancer Biomarkers

**DOI:** 10.3390/diagnostics14060609

**Published:** 2024-03-13

**Authors:** Ugur Gezer, Angela Oberhofer, Karolina Worf, Oliver Stoetzer, Stefan Holdenrieder, Abel Bronkhorst

**Affiliations:** 1 Department of Basic Oncology, Oncology Institute, Istanbul University, Istanbul 34093, Türkiye; ugurd@istanbul.edu.tr; 2 Munich Biomarker Research Center, Institute of Laboratory Medicine, German Heart Center, Technical University Munich, 80636 Munich, Germany; oberhofer@dhm.mhn.de (A.O.); karolina.worf@tum.de (K.W.); holdenrieder@dhm.mhn.de (S.H.); 3 Medical Center for Hematology and Oncology Munich GmbH, 80639 Munich, Germany; ojstoetzer@aol.com

**Keywords:** cancer, liquid biopsy, ctDNA, repetitive DNA, human satellite 2, targeted sequencing

## Abstract

Liquid biopsies are revolutionizing the detection and management of malignant diseases. While repetitive DNA sequences, such as LINE-1 and ALU are established in cell-free DNA (cfDNA) research, their clinical applications remain limited. In this study, we explore human satellite 2 (HSATII), a prevalent repeat DNA sequence in plasma that exhibits increased levels in cancer patients, thereby positioning it as a potential pan-cancer biomarker. We employed targeted sequencing and copy number variation (CNV) analysis using two primer pairs to assess the differential abundance of HSATII sequences in the plasma of breast cancer patients compared to healthy individuals. PCR amplicons of HSATII from 10 patients and 10 control subjects were sequenced, generating 151 bp paired-end reads. By constructing a pooled reference dataset, HSATII copy ratios were estimated in the patients. Our analysis revealed several significant CNVs in HSATII, with certain sequences displaying notable gains and losses across all breast cancer patients, suggesting their potential as biomarkers. However, we observed pronounced fragmentation of cfDNA in cancer, leading to the loss of longer PCR amplicons (>180 bp). While not all observed losses can be attributed to fragmentation artifacts, this phenomenon does introduce complexity in interpreting CNV data. Notably, this research marks the first instance of targeted HSATII sequencing in a liquid biopsy context. Our findings lay the groundwork for developing sequencing-based assays to detect differentially represented HSATII sequences, potentially advancing the field of minimally-invasive cancer screening.

## 1. Introduction

Liquid biopsy or liquid profiling refers to the analysis of tumor-derived biomarkers in body fluids. These markers include circulating tumor cells, various genetic and epigenetic features of circulating tumor DNA (ctDNA), cell-free DNA (cfDNA), and cell-free RNA (cfRNA), extracellular vesicles, proteins, metabolites, and others. Of these, ctDNA analysis, which is based on the detection of tumor-derived mutations in plasma DNA, possesses the broadest application in oncology (reviewed in [1,2,3,4]), including the detection of minimal residual disease [5], earlier detection of recurrence [6,7], guiding the selection of targeted therapies [8], treatment monitoring [9], detection of resistance mechanisms [10], and prognosis [11].

Liquid biopsy assays, harnessing the detection of ctDNA, are progressively being integrated into standard cancer care. This integration is poised to fundamentally transform oncological practices in the foreseeable future, as highlighted in the recent literature [1,2,3,4]. Despite their potential, the development of diagnostic tests that achieve the requisite sensitivity and specificity for widespread application in routine clinical practice presents significant challenges. A primary obstacle in ctDNA analysis is the often-minimal presence of cancer-associated point mutations, particularly in the early stages of the disease. This low abundance poses a substantial hurdle to detecting ctDNA effectively. To enhance sensitivity in ctDNA detection, one promising approach is to broaden the spectrum of ctDNA characteristics under examination. Beyond the conventional focus on hotspot mutations, more comprehensive profiling that includes a range of genetic and epigenetic features is proposed. This approach encompasses examining broader-scale phenomena across the cancer genome, such as DNA methylation patterns, histone modifications, cfDNA fragmentation profiles, nucleosome occupancy patterns, and the presence of aberrant repetitive DNA sequences [12,13,14,15,16,17]. Our current study centers on the latter—repetitive DNA—which is a significant yet often overlooked component of the human genome, constituting more than 50% of its total content [15,18]. This abundance is mirrored in cfDNA [15,19,20], making repetitive DNA an intriguing target for overcoming some of the technical challenges inherent in traditional ctDNA assays. Among these repetitive sequences, LINE-1 and ALU elements, two typical representatives of long interspersed nuclear elements (LINEs) or short interspersed nuclear elements (SINEs) [21], have garnered substantial attention in liquid biopsy research. These elements have been utilized in various capacities, including the quantification of total cfDNA levels [22,23], the analysis of cfDNA integrity [24,25,26], the detection of aneuploidy [27,28] and microsatellite alterations in cfDNA [29], and the assessment of aberrant DNA methylation in cfDNA [30]. Despite the wide-spread application and detection of many cancer-associated alterations using these elements, the lack of cancer specificity limits their use in the management of cancer patients.

Satellite DNA represents one of the most fascinating parts of the repetitive fraction of the eukaryotic genome, albeit underrepresented in genomic studies [31]. Centromeric/pericentric satellite sequences are constituents of centromeric and pericentromeric heterochromatin and have been implicated in chromosome organization and segregation, kinetochore formation, as well as heterochromatin regulation [32]. Human satellite 2 (HSATII), a typical pericentric satellite repeat including repeating units of the pentameric CATTC repeat over long arrays on given chromosomes, has been shown to expand in copy number in cancer cells by the incorporation of HSATII-RNA-derived DNA molecules within pericentromeric loci [33]. On the other hand, next-generation sequencing of cfDNA demonstrated another intriguing trait of these elements: compared to their genomic abundance, pericentric satellites were found to be the most overrepresented repeat (2230%) in the cfDNA population in healthy individuals [19]. Overrepresentation in cfDNA in physiologic conditions and copy number expansion in tumor cells make pericentric satellite repeats a promising biomarker for liquid biopsy in patients with cancer. In line with this potential, in a recent exploratory study, we found significantly higher levels of HSATII repeat DNA in the plasma of cancer patients vs. healthy subjects [34], where the primer set for chromosome 10-related HSATII repeat DNA (Chr10-HSATII) resulted in better discrimination between cancer patients and healthy subjects. Here, we hypothesized that different HSATII sequences in the pericentric region of chromosomes may be differentially expressed between the blood samples of cancer patients and healthy subjects. In addition, depending on the primer binding sites, the fragmentation level of plasma DNA could affect some HSATII-derived amplicons, as it is well documented that in contrast to healthy cfDNA with a homogeneous fragmentation pattern, patients with cancer show several distinct genomic differences with increases and decreases in the fragment sizes of cfDNA at different regions [35]. Thus, via targeted amplicon sequencing, we aimed to evaluate the abundance of various HSATII sequences in the plasma of cancer patients.

## 2. Materials and Methods

### 2.1. Patients and Plasma DNA Extraction

Archived plasma samples from metastatic breast cancer patients (*n* = 10) and healthy controls (*n* = 10) were collected for an earlier biomarker study at the Hematology/Oncology Speciality Center Munich and stored at −80 °C at the LMU University Hospital Munich (approved by LMU Ethics Commission Project-Nr. 183-08). These samples were used in an anonymized way for the identification of diagnostic oncological plasma biomarkers, a practice permitted by LMU Ethics Commission UE Nr. 111-14, and then subsequently enrolled in the present pilot study. cfDNA was extracted from 500 µL of plasma from both the healthy subjects and cancer patients using the QIAamp MinElute cfDNA Mini Kit (Qiagen, Hilden, Germany) according to the manufacturer’s instructions. The cfDNA quantity and quality were assessed with the Agilent Bioanalyzer High Sensitivity (HS) DNA assay (Agilent, Santa Clara, CA, USA).

### 2.2. Amplification of HSATII Repeat Sequences from Plasma DNA

**Figure 1:**
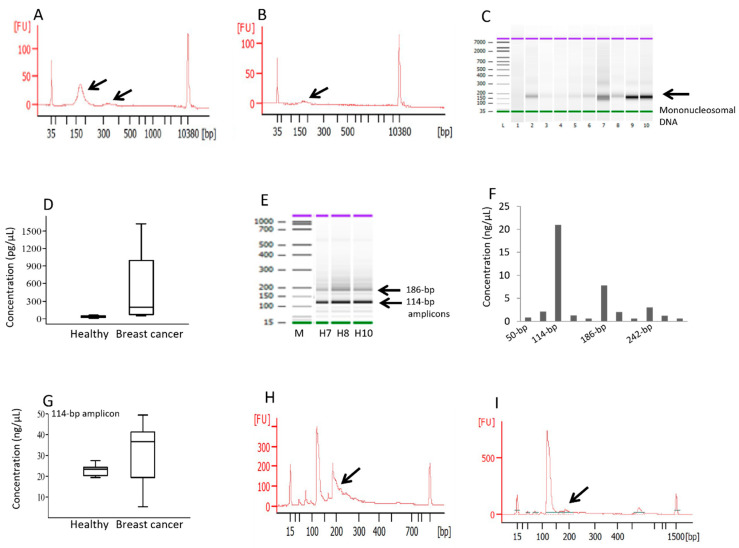
Fragmentation analysis of cfDNA and size analysis of Chr10-HSATII amplicons. The total cfDNA extracted from the samples of the patients (**A**) and the control subjects (**B**) was separated via automated electrophoresis, with the arrows pointing to mono-nucleosomal and di-nucleosomal DNA at around 180 and 360 bp. These are representative figures; the size profiles of all patients and control subjects can be seen in Supplementary Figure S1A,B, respectively. (**C**) Gel-like display of the separated cfDNA in which mono-nucleosomal DNA in varying amounts among the patient samples is highlighted by an arrow. (**D**) Concentrations of mono-nucleosomal cfDNA peaks in both the healthy subjects and the patients, with the median, lowest, and highest values. Following PCR amplification, HSATII amplicons were analyzed by automated electrophoresis. (**E**) Gel-like display of the HSATII amplicons in three control subjects with the 114 bp amplicon as the predominant product, highlighted by an arrow. (**F**) Concentrations of individual PCR amplicons in a representative healthy control sample. (**G**) The concentrations of the 114 bp amplicon in both the healthy subjects and the patients. (**H**,**I**) The size distribution of the Chr10-HSATII amplicons in two representative samples of the controls and patients, respectively. The arrows indicate amplicons longer than 180 bp. The concentrations of mono-nucleosomes and amplicons were determined using the built-in size-gating function of the Agilent Bioanalyzer 2100 software. The size distribution of the Chr10-HSATII amplicons for all patients and control subjects can be seen in Supplementary Figures S2A and S2B, respectively. Size analysis of the Chr1-HSATII amplicons for all patients and control subjects can be seen in Supplementary Figures S3A and S3B, respectively.

We amplified HSATII repeat sequences using two primer pairs that amplify their target regions typically located in the pericentric regions of Chr1 (Chr1-HSATII) and Chr10 (Chr10-HSATII). The primer sequences used for Chr1 were 5′-GGTTCCATTTGATGATGATTCC-3′ (forward) and 5′-ATCGAGTGGAATCGAATGG-3′ (reverse). The primer sequences used for Chr10 were 5′-GACGATTCCATTCACTTCC-3′ (forward) and 5′-GAAAGGAGTCATCATCTAATGG-3′ (reverse). PCR was performed with the Taq PCR kit (New England Biolabs, Ipswich, MA, USA) as previously reported [33]. PCR products were purified with the Monarch PCR & DNA Cleanup Kit (New England Biolabs) and visualized with the Bioanalyzer DNA 1000 assay (Agilent). As primers bind to several target sequences, PCR amplification using the Chr1-HSATII primer set resulted in a maximum of 12 different amplicons ranging from 43–325 bp in size with 144 bp as the main product (Supplementary Figure S3A,B). PCR amplification using the Chr10-HSATII primer set resulted in a maximum of 12 different amplicons ranging from 50 to 559 bp in size with the 114 bp fragment as the main product (approx. 50% of all amplicons in concentration), as visualized on the automated electrophoresis system (Figure 1E,F). In one sample (Patient 8), the concentration of the 114 bp band was much lower than that of the other patient and control samples.

### 2.3. Sequencing of HSATII Amplicons

Purified amplicons served as the starting material for library construction. Separate libraries were constructed for each respective patient and control sample, with specific primer sets for both Chr1 and Chr10. The total concentrations of amplicons were taken into consideration for the library preparations, and equal amounts of each individual library were pooled for sequencing. Library preparation was performed with the NEBNext Ultra II DNA Library Prep Kit (New England Biolabs) and NEBNext Multiplex Oligo (Index Primers Set 1 and 2; New England Biolabs) according to the manufacturer’s instructions for standard DNA without size selection. Before sequencing, the library was purified and checked on the Agilent Bioanalyzer. After purification, likely as a consequence of enrichment, new amplicons emerged (about 35 in total), and most of them were in negligible quantities. The input libraries were normalized among the samples by setting the concentrations to 10 nM. Sequencing was performed with the MiniSeq instrument (Illumina, San Diego, CA, USA) using the MiniSeq Mid Output (300 cycles) reagent kit (Illumina) with 2x 151 bp paired-end reads. The sequencing quality control metrics were as follows: Q30—the percentage of bases with a quality score of 30 or higher was 87.4%; the density of clusters on the flow cell (K/mm^2^) was 125; and finally, the percentage of clusters that passed the filter (%PF) was 78.9%. As expected, the reads were aligned to chromosomes 1 and 10, but also to chromosomes 2, 7, and 16. The latter was expected due to the high sequence similarity of repetitive DNA across the chromosomes.

### 2.4. Bioinformatic Analysis

To assess the sequencing quality and identify potential errors, quality control of the raw sequencing reads was performed with FastQC [36], fastp [37], and MultiQC [38]. Adapter sequences identified in the sequencing data, including the forward adapter 5′-AGATCGGAAGAGCACACGTCTGAACTCCAGTCA-3′ and the reverse adapter 5′-AGATCGGAAGAGCGTCGTGTAGGGAAAGAGTGT-3′, along with automatically detected adapters, were trimmed. Quality filtering was then applied, setting thresholds for a minimum read length of ≥50, a quality score of ≥20, and a limit of 50% for the proportion of unqualified bases per read. Additionally, sequencing artefacts (first 20 bp at 3′ ends due to low quality) were eliminated with cutadapt [39] and fastp, since neither tool alone could remove all identified adapters and artefacts. Burrows–Wheeler aligner (BWA) [40] was applied to align the remaining sequencing reads to the human reference genome hg38.p14 (GRCh38.p14) downloaded from the UCSC Genome Browser [41]. The aligned sequencing reads in SAM format were converted to BAM, coordinate-sorted, and indexed using Samtools [42]. CNVkit’s [43] targeted amplicon sequencing (TAS) command line was used to estimate the copy ratios of HSATII in the plasma of the breast cancer patients. Thus, the BAM files of the control samples, the FASTA file of the hg38 reference genome, and the BED file of the target intervals were specified, and low coverage bins were dropped. Since the original BED file of the target intervals was not available, it was generated as follows: (1) The unguided mode of CNVkit’s guess_baits.py script was used with all sample BAM files and the 10 kb accessibility file of hg38, followed by (2) CNVkit’s target command with the newly generated baits BED file, the refFlat file of hg38, and the --split option to obtain equal-sized regions of the targets. The sequencing read depths of the patient samples were normalized and compared individually to a pooled reference of the 10 healthy control samples. The copy ratios for the patients were defined as the log2 ratio between the observed and expected number of reads in the target intervals. Thus, the log2 fold change always represents the patients against the pooled control data. Regions bearing a log2 ratio of at least ±0.4 were considered as suggestive of shallow copy losses or gains [43]. Log2 ratios up to −1.1 were assigned a copy number of 0, whereas those with log2 ratios >1.1 indicate multiple excess copies. In addition to the log2 copy ratios, we considered the ‘weight’ values, which show each bin’s proportional weight or reliability mainly derived from the inverse of the variance of the normalized log2 coverage values in all control samples at that bin. In the CNVkit pipeline, the determination of the weights and bin sizes was automated to gauge the reliability of the genomic bins, an important aspect for the precise analysis of CNVs. These weights were computed based on various factors, encompassing the bin’s size relative to the average, deviations from neutral coverage for paired or pooled references, and the inverse variance of the normalized log2 coverage values for the pooled references. Consequently, bins assigned higher weights are considered more dependable and exert greater influence on the bin log2 ratio values during segmentation, thereby enhancing the accuracy of CNV detection. In CNVkit’s visualization, particularly with the “scatter” command, the size of the plotted data points is directly proportional to each bin’s weight, simplifying the identification of regions harboring potentially less reliable data—where smaller points indicate lower reliability.

### 2.5. Statistical Analysis and Data Visualization

R Statistical Software (v4.2.1) [44] and Excel (v3.4.4.) were utilized for all statistical analyses and data visualizations. The cor.test function in R was used to calculate the Pearson correlation and the p-value between the weighted mean log2 copy ratio and the weighted mean log2 read depth of all patients per target region. Data visualization was performed with *ggplot2* version 3.4.4 [45] in R.

## 3. Results

### 3.1. High Fragmentation of cfDNA in Breast Cancer Patients Affects the Amplification of Longer HSATII Amplicons

Automated electrophoresis of the cfDNA showed that mono-nucleosomal DNA (~180 bp) constituted the main cfDNA population in cancer patients (Figure 1A, Supplementary Figure S1A) compared to the cfDNA of healthy subjects, with less pronounced peaks (Figure 1B, Supplementary Figure S1B). Di-nucleosomes were detected in a small number of patients (Supplementary Figure S1A). Although the concentrations of mono-nucleosomal cfDNA were highly variable among the patient samples (Figure 1C), the patients as a group exhibited significantly higher levels than the healthy subjects (Figure 1D, median 190 pg/µL vs. 24 pg/µL, respectively). Automated electrophoresis of the Chr10-PCR amplicons revealed the 114 bp amplicons as the predominant product (Figure 1E), constituting ~50% of the total concentration (Figure 1F).

The abundance of 114 bp amplicons were considerably higher in the breast cancer patients than in the healthy subjects (Figure 1G, medians of 24 ng/µL vs. 38 ng/µL, respectively). It is evident that the majority of Chr10 amplicons longer than 180 bp were missing from the patient samples (Figure 1I, Supplementary Figure S2A) compared to the control samples (Figure 1H, Supplementary Figure S2B), likely as a consequence of increased DNA fragmentation. The automated electrophoresis results for the Chr1 amplicons are shown in Supplementary Figure S3A,B. Interestingly, the loss of amplicons larger than ~180 bp was much less pronounced for Chr1 amplicons vs. Chr10 amplicons, likely due to their lower proportion.

### 3.2. Estimation of Copy Ratios of HSATII in Breast Cancer Patients

**Figure 2:**
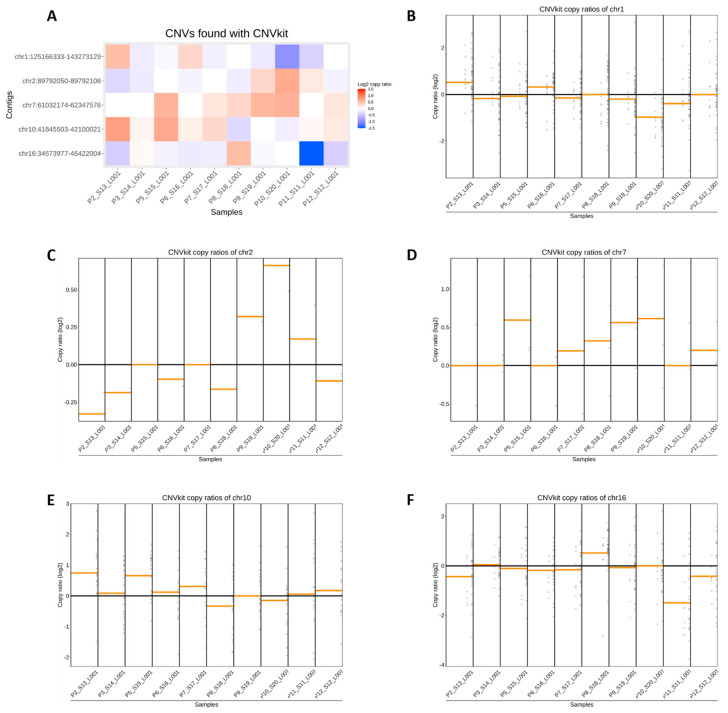
Visualization of HSATII sequence CNVs in cfDNA. (**A**) Heatmap representing relative copy ratio alterations of HSATII sequences in patient cfDNA samples compared to a pooled reference from healthy individuals, with cooler colors (blue) indicating a potential loss of amplicons, and warmer colors (red) denoting an increase in copy ratios within the plasma of patients. (**B**–**F**) The individual plots display the copy ratios for specific HSATII sequences and the computed average copy ratios for each patient across chromosomes 1, 2, 7, 10, and 16, respectively. The horizontal axis lists the patient samples, while the vertical axis quantifies the copy ratio changes. Each plot shows a threshold line at a copy ratio of 0, with deviations above and below this line suggesting relative gains and losses in the copy number.

In this study, we employed the CNVkit pipeline to analyze CNVs in HSATII repeat sequences in the cfDNA of breast cancer patients. We identified 101 conserved sequences across chromosomes 1, 2, 7, 10, and 16 in all patient samples (the sequences can be found in Supplementary Table S1). These sequences displayed considerable variability in their read depth (ranging from 1.56 to 4961, with an average of approximately 283), log2 copy ratios (ranging from 3.09 to −3.16, with an average of −0.066), and weight values (ranging from 0 to 0.97, with an average of 0.56) (Supplementary Table S2). This variability suggests a differential abundance of these sequences within the cfDNA. Our analysis identified a balanced distribution of copy number gains (482 instances, average gain of +0.49) and losses (512 instances, average loss of −0.51) across the patient cohort (Supplementary Table S3). Figure 2 illustrates the average CNVs within each chromosome per patient. Notably, chromosomes 7 and 10 predominantly exhibited copy number gains, whereas chromosomes 1, 2, and 16 showed more frequent losses. However, individual patient analyses revealed a wide range of CNVs within each chromosome, indicating substantial heterogeneity in amplicon alterations (Figure 2B–F). Linear regression analysis correlating log2 copy ratios with log2 read depths across the 101 sequences revealed a moderate mean correlation coefficient (r = 0.6). This correlation, however, was not uniformly distributed: approximately 52% of the sequences demonstrated strong correlations (r > 0.7), and 85% exhibited moderate-to-strong correlations (r > 0.4) (Supplementary Table S3). Conversely, no significant correlation was observed between the read depth and weight (r = −0.006976), nor between the log2 copy ratios and sequence weight (r = −0.11). Interestingly, an analysis of the extremes revealed that the 10 sequences with the strongest correlation between the log2 copy ratios and the log2 read depths had an average weight of 0.43, in contrast to an average weight of 0.66 for the 10 sequences with the weakest correlation. Table 1 summarizes the 10 sequences with the most significant copy number gains and losses. However, our results suggest that while several amplicons demonstrate notable copy number changes, those consistently occurring across all patients may hold the highest potential as biomarkers. Among the 101 common sequences, 3 showed gains and 4 showed losses in all patients. Additionally, eight sequences exhibited gains in all but one patient, and seven showed losses under similar conditions (Table 2). Particularly relevant as potential cancer biomarkers are those sequences that demonstrate significant copy number changes, robust weight values, and a linear relationship between the copy ratio and read depth. For instance, the amplicon at Chr10:42099756-42099907, despite a high log2 copy ratio (−1.48), exhibited a low weight value (0.17), indicating high variance among the control samples, and thus, reduced reliability. In contrast, the amplicon at Chr1:143235834-143235977, while showing a slightly lower log2 copy ratio (−1.38), had a higher weight value (0.77), suggesting greater potential as a biomarker.

However, it is important to note that the increased fragmentation of cfDNA in cancer patients and the subsequent loss of longer PCR amplicons in the cancer patients vs. healthy subjects (Figure 1H,I and Figure S2A,B) can influence CNV analysis and interpretation. This fragmentation limits the amplification of longer DNA sequences, often exceeding 180 base pairs, leading to potential underrepresentation or the omission of crucial genomic regions in the analysis. Consequently, this can result in apparent losses of genetic material in sequencing data, manifesting as ‘losses’ in HeatMaps and potentially leading to inaccuracies in CNV interpretation. Furthermore, this fragmentation impacts the correlation between read depths and log2 copy ratios, with variability among control samples potentially compromising the reliability of high-read-depth sequences. These challenges extend to the accurate identification of biomarkers, as the fragmented nature of cfDNA can hinder the clear differentiation between cancerous and non-cancerous samples.

## 4. Discussion

Building on insights from our previous exploratory study [34], we identified HSATII repeat DNA as a promising pan-cancer plasma biomarker for liquid biopsy. This recognition stems from our observation of elevated HSATII levels in the plasma of cancer patients. The HSATII repeat, characterized by its presence in variable copy numbers throughout the genome, poses a unique analytical challenge. Certain HSATII-derived amplicons are susceptible to fragmentation in cfDNA, leading to variability in their representation. Consequently, different HSATII sequences, particularly those from diverse sub-genomic regions, may have varying capacities to distinguish between cancerous and non-cancerous conditions. This guided us to focus specifically on the HSATII sequences associated primarily with chromosomes 1 and 10. By targeting these sequences, we aimed to discern their unique contribution to cancer detection, exploring how their variability impacts their utility as biomarkers. To achieve this, we employed targeted amplicon sequencing, a method selected for its distinct advantages over whole-genome sequencing in this context. Targeted amplicon sequencing offers a more cost-effective approach, simplifies data complexity, and importantly, provides higher coverage of the targeted regions. This focused approach allowed for a more detailed examination of the specific HSATII sequences, enhancing our ability to accurately assess their potential as diagnostic markers in cancer [46].

Pre-sequencing analysis revealed increased fragmentation of plasma cfDNA in metastatic breast cancer patients vs. healthy subjects, as is commonly reported for cfDNA in cancer patients of different tumor types [47,48]. Mono-nucleosomes constituted the main fraction of cfDNA in the majority of the patients’ samples, while di-nucleosomes were observed in some samples at lower levels, but not in the healthy subjects. In line with this, in a recent report employing single- and double-strand sequencing of cfDNA, the proportion of mono-nucleosomes, di-nucleosomes, and larger fragments (>1000 bp) were estimated to be 67.5% to 80%, 9.4% to 11.5%, and 8.5% to 21.0%, respectively [47]. In healthy individuals, the size profile of cfDNA fragments is more homogenous than in cancer patients [35,47]. This differential size profile of plasma DNA between cancer patients and non-cancer conditions could affect amplification-based cfDNA measurements in liquid profiling, and was observed in our study, where the Chr10-amplicons larger than 180 bp were mostly absent in the cancer patients and manifested as copy losses in the HeatMap (Figure 2A).

While it is unlikely that all losses are artifacts, given the complexity of cancer genomics and cfDNA analysis, it is possible that a significant portion of the observed losses could be influenced by cfDNA fragmentation. This could lead to inaccuracies in identifying potential biomarkers and understanding the genomic landscape of breast cancer. Given these potential issues, the findings need to be validated with additional methods, such as long-read sequencing, to confirm whether the observed CNV losses are genuine or artifacts of the analysis method. Nevertheless, this indicates that, depending on the selection of primers and their binding sites, the choice of larger amplicons could introduce bias to the outcome measures. Thus, any assay development based on HSATII quantification in liquid profiling will largely depend on the primer selection, also considering cfDNA fragmentation. In CNV analysis of tumor samples, the relationship between the observed copy ratios and the true copy number is not linear, as bulk tumor samples are mixtures of billions of cells, including normal ones [49]. The same is true for plasma, as the fraction of DNA derived from tumor cells is low, sometimes less than 0.01% [50,51]. Thus, the total cfDNA concentration measured in the blood is not a reliable biomarker for tumor burden and is subject to significant technical and biological variability [52].

## 5. Conclusions

Our results indicate that HSATII sequences are present in significantly higher quantities in the plasma of cancer patients compared to control subjects. We observed a positive correlation between the coverage depths and log2 copy ratios, suggesting that sequences with greater plasma abundance are more promising as target sequences for cancer detection. However, not all high-depth sequences are equally relevant. The variability in abundance across control samples impacts the reliability of some sequences, indicating that they may not be suitable biomarkers. This study is pioneering in proposing that the targeted sequencing of HSATII sequences could be instrumental in distinguishing cancerous conditions from non-malignant ones. Given its exploratory nature, this study was conducted with a limited sample size. If future research involving larger sample sizes and various tumor types confirms these findings, HSATII DNA sequences could potentially be developed into PCR assays. These assays might serve as effective pan-cancer screening tools, offering a new avenue for cancer management.

## Figures and Tables

**Table 1:** Significant copy number variations across all patients, sorted by log2 copy ratios.

	Chromosomal Coordinates	Log2 Copy Ratio	Weight	Log2 Copy Ratio vs. Log2 Read Depth (Pearson’s r)
Significant copy number gains	Chr1:143234645-143234759	1.98	0.29	0.65
	Chr16:46395162-46395250	1.32	0.57	0.21
	Chr16:46398850-46398995	1.22	0.42	0.71
	Chr10:42087568-42087682	1.19	0.43	0.47
	Chr10:41850696-41850784	1.08	0.66	0.74
	Chr1:125173758-125173872	1.02	0.85	0.40
	Chr10:41845503-41845591	0.88	0.35	0.74
	Chr1:143265528-143265702	0.78	0.49	−0.19
	Chr10:42093679-42093822	0.73	0.34	0.77
	Chr1:143249938-143250076	0.65	0.74	0.60
Significant copy number losses	Chr16:34594210-34594279	−0.96	0.31	0.85
	Chr10:42097372-42097590	−0.96	0.93	0.87
	Chr16:34591868-34591971	−1.03	0.35	0.91
	Chr16:46394749-46394886	−1.16	0.39	0.79
	Chr1:143256460-143256641	−1.24	0.56	−0.06
	Chr1:143206616-143206759	−1.29	0.63	−0.68
	Chr16:46390193-46390340	−1.38	0.77	0.82
	Chr16:46388855-46388979	−1.48	0.17	0.83
	Chr1:143235834-143235977	−1.50	0.23	0.46
	Chr16:34591728-34591842	−1.58	0.87	−0.19

**Table 2:** Significant copy number variations across all patients, sorted by frequency of occurrence.

	Chromosomal Coordinates	Total Gains	Total Losses	Log2 Copy Ratio		Weight		Copy Ratio vs. Read Depth Correlation (Pearson’s r)
				Avg.	Std. Dev.	Avg.	Std. Dev.	
Significant copy number gains	Chr16:46395162-46395250	10	0	1.32	0.51	0.57	0.03	0.21
	Chr16:46398850-46398995	10	0	1.22	0.37	0.42	0.02	0.71
	Chr16:46421913-46422004	10	0	0.49	0.33	0.76	0.03	0.61
	Chr1:143234645-143234759	9	1	1.98	1.03	0.29	0.02	0.65
	Chr10:42087568-42087682	9	1	1.19	0.84	0.43	0.02	0.47
	Chr10:41850696-41850784	9	1	1.08	1.01	0.66	0.03	0.74
	Chr10:41845503-41845591	9	1	0.88	1.17	0.35	0.03	0.74
	Chr1:143265528-143265702	9	1	0.78	0.63	0.49	0.02	−0.19
	Chr1:143244455-143244541	9	1	0.64	0.47	0.27	0.03	0.49
	Chr16:34588502-34588580	9	1	0.58	0.38	0.70	0.03	0.77
	Chr16:46389286-46389443	9	1	0.56	0.38	0.43	0.02	0.71
Significant copy number losses	Chr16:46394749-46394886	1	9	−0.63	0.77	0.66	0.02	0.64
	Chr1:125166914-125167051	1	9	−0.64	0.53	0.20	0.02	0.89
	Chr16:34573977-34574077	1	9	−0.68	0.89	0.32	0.02	0.73
	Chr1:143239360-143239536	1	9	−0.74	0.86	0.46	0.02	0.20
	Chr16:34591868-34591971	1	9	−0.78	0.77	0.52	0.02	0.87
	Chr16:46390193-46390340	1	9	−1.29	0.85	0.63	0.02	−0.68
	Chr16:34591728-34591842	1	9	−1.58	1.02	0.87	0.02	−0.19
	Chr16:46390975-46391112	0	10	−0.96	0.47	0.31	0.02	0.85
	Chr1:143206616-143206759	0	10	−1.24	0.77	0.56	0.02	−0.06
	Chr1:143235834-143235977	0	10	−1.38	0.55	0.77	0.02	0.82
	Chr10:42099756-42099907	0	10	−1.48	0.38	0.17	0.02	0.83

## Data Availability

The authors confirm that the data supporting the findings of this study are available within the article and will be provided upon reasonable request.

## Supplementary Materials

The following supporting information can be downloaded at: https://www.mdpi.com/article/10.3390/diagnostics14060609/s1, Figure S1: (A) Automated electrophoresis of total plasma cfDNA of breast cancer patients; (B) Automated electrophoresis of total plasma cfDNA of healthy subjects. Figure S2: (A) Automated electrophoresis of Chr10-HSATII PCR amplicons in patients’ samples; (B) Automated electrophoresis of Chr10-HSATII PCR amplicons in control samples. Figure S3: (A) Automated electrophoresis of Chr1-HSATII PCR amplicons in patients’ samples; (B) Automated electrophoresis of Chr1-HSATII PCR amplicons in control samples. Table S1: DNA_sequence; Table S2: Combined data; Table S3: Contig gains sorted.
