# Supplementary material for: Targeted Sequencing of Human Satellite 2 Repeat Sequences in Plasma cfDNA Reveals Potential Breast Cancer Biomarkers
Source: Diagnostics (Basel). 2024 Mar 13;14(6):609. doi: 10.3390/diagnostics14060609 (PMC10968943; doi:10.3390/diagnostics14060609)
Supplement: Supplementary file 1 [file diagnostics-14-00609-s001.zip › Supplementary Figures.pdf]

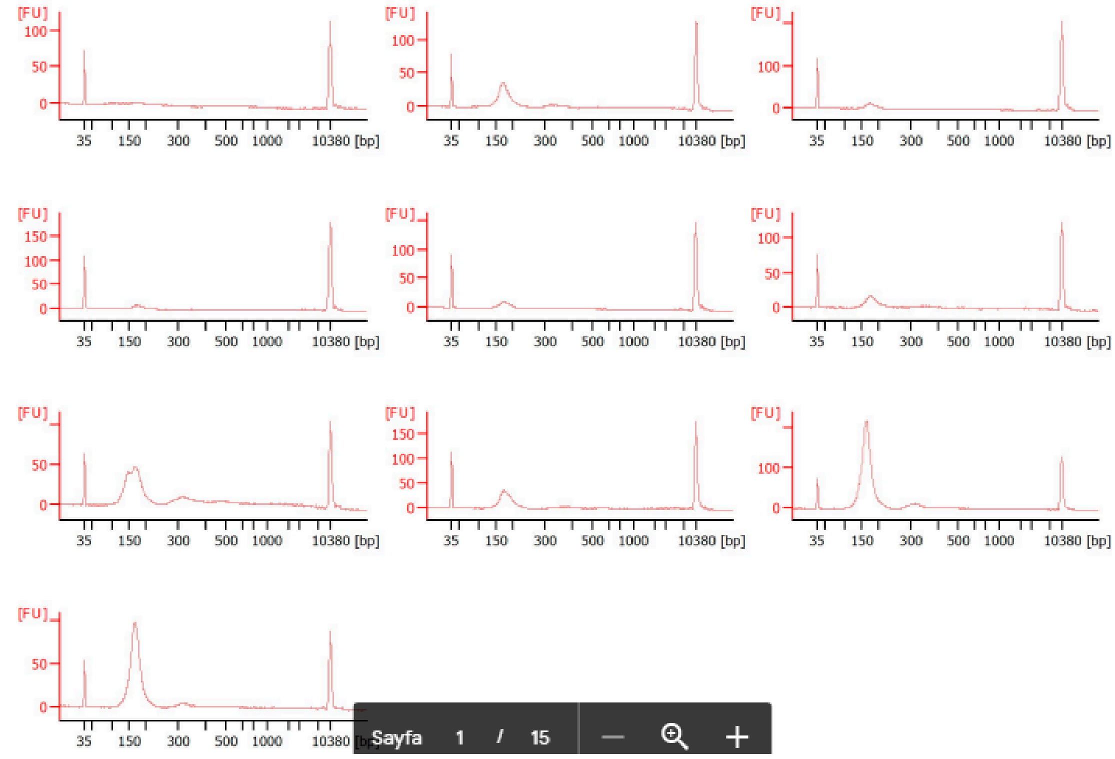


**Figure S1A:** Automated electrophoresis of total plasma cfDNA of breast cancer patients

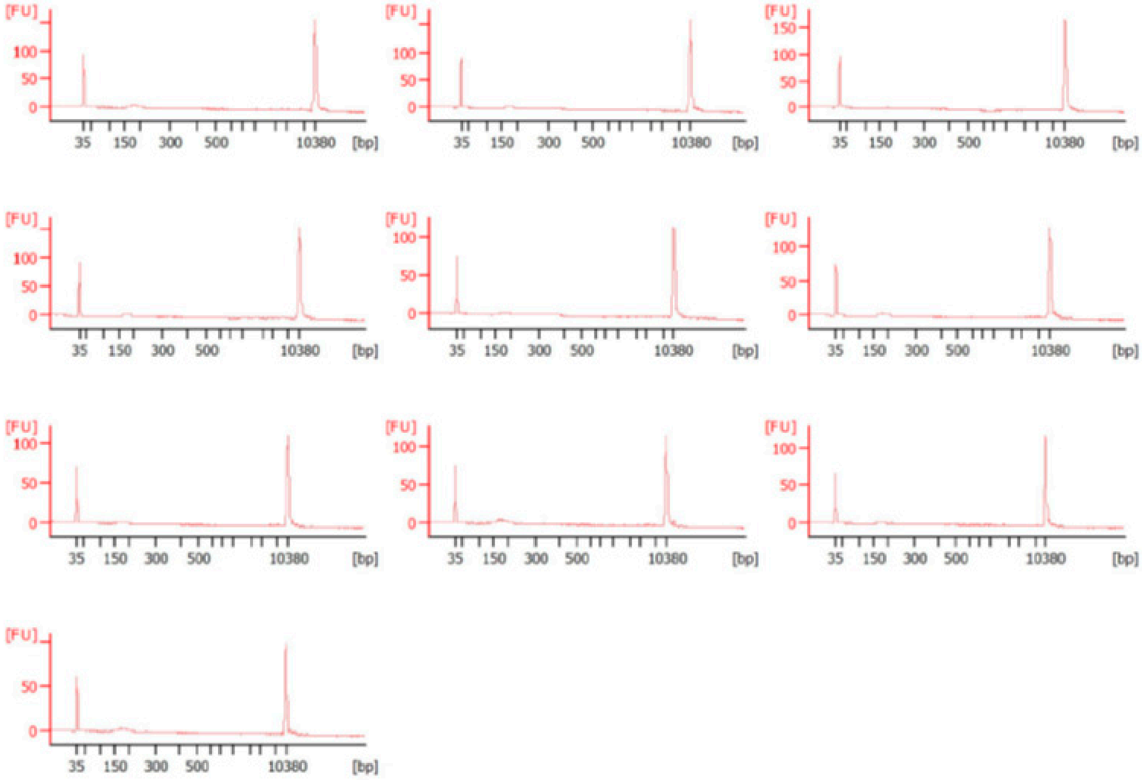


**Figure S1B**. Automated electrophoresis of total plasma cfDNA of healthy subjects

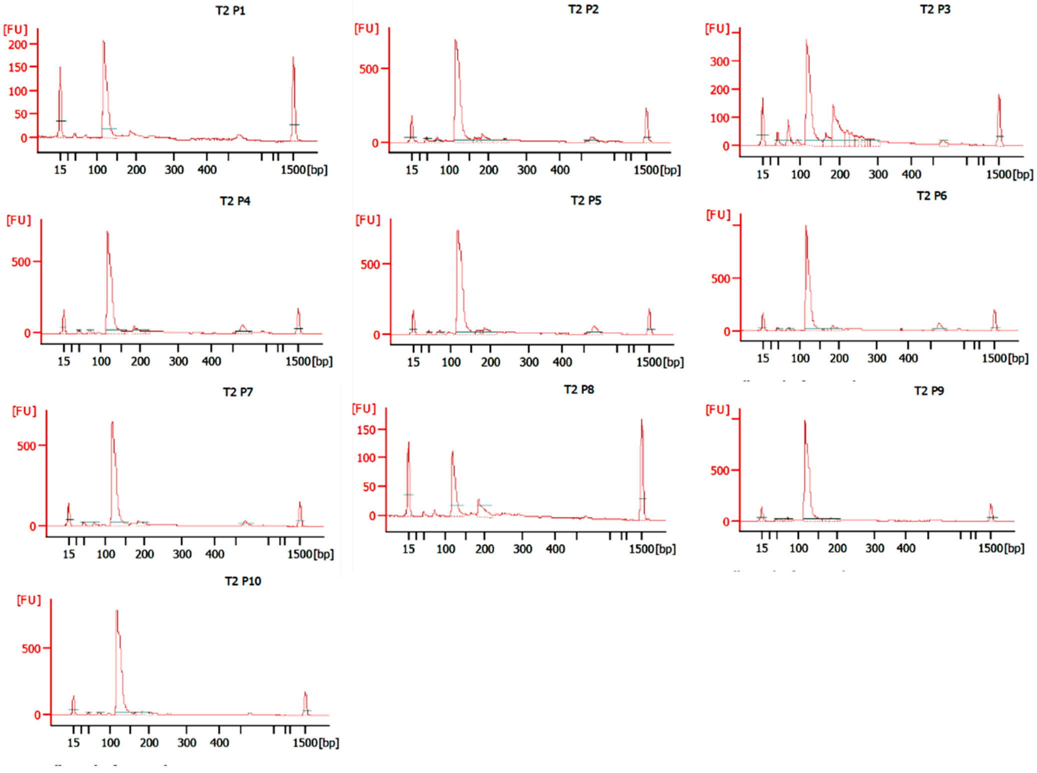


**Figure S2A.** Automated electrophoresis of Chr10-HSATII PCR amplicons in patients' samples

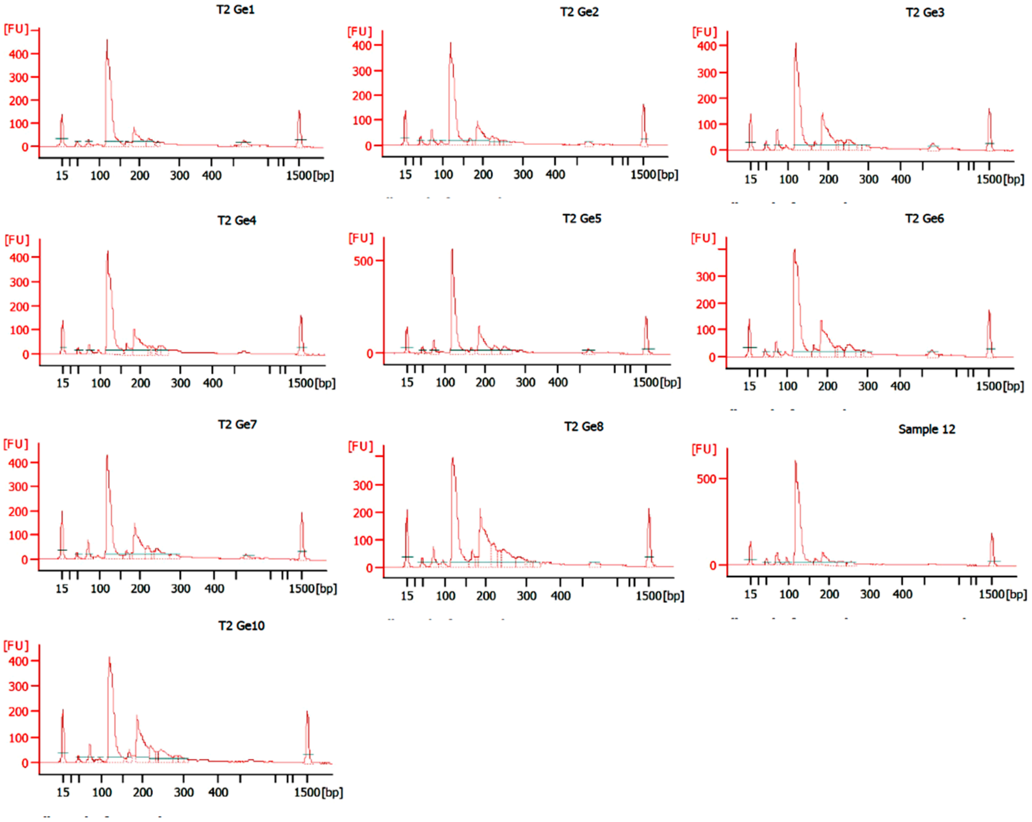


**Figure S2B.** Automated electrophoresis of Chr10-HSATII PCR amplicons in control samples

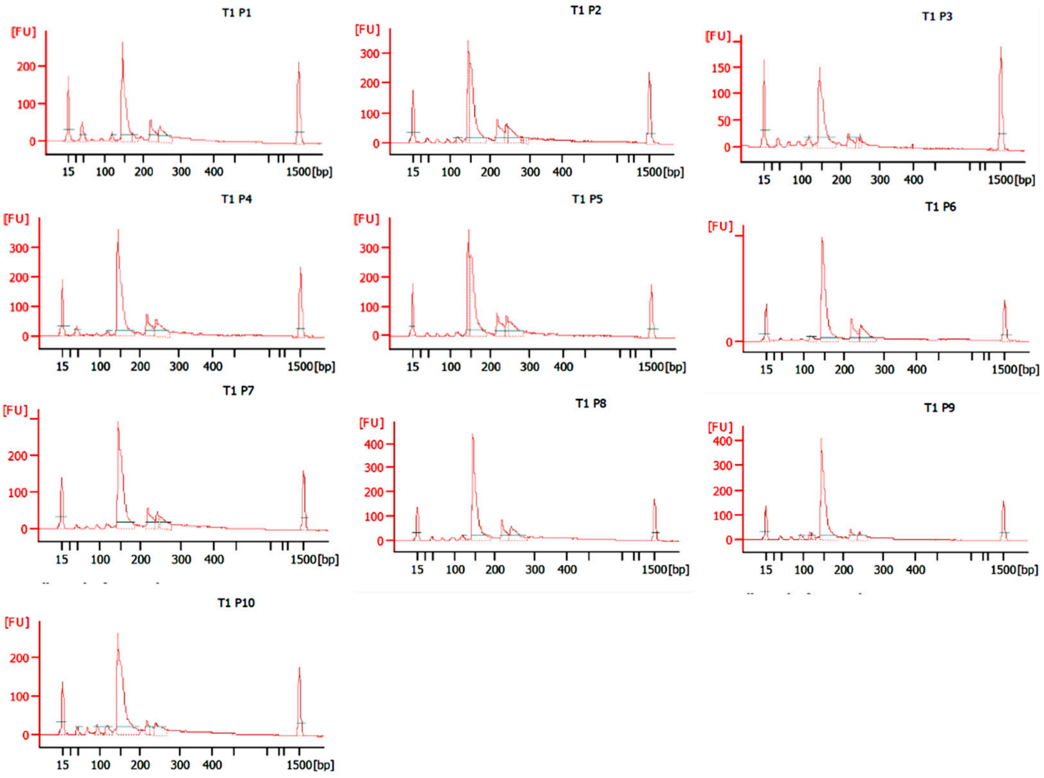


**Figure S3A.** Automated electrophoresis of Chr1-HSATII PCR amplicons in patients' samples

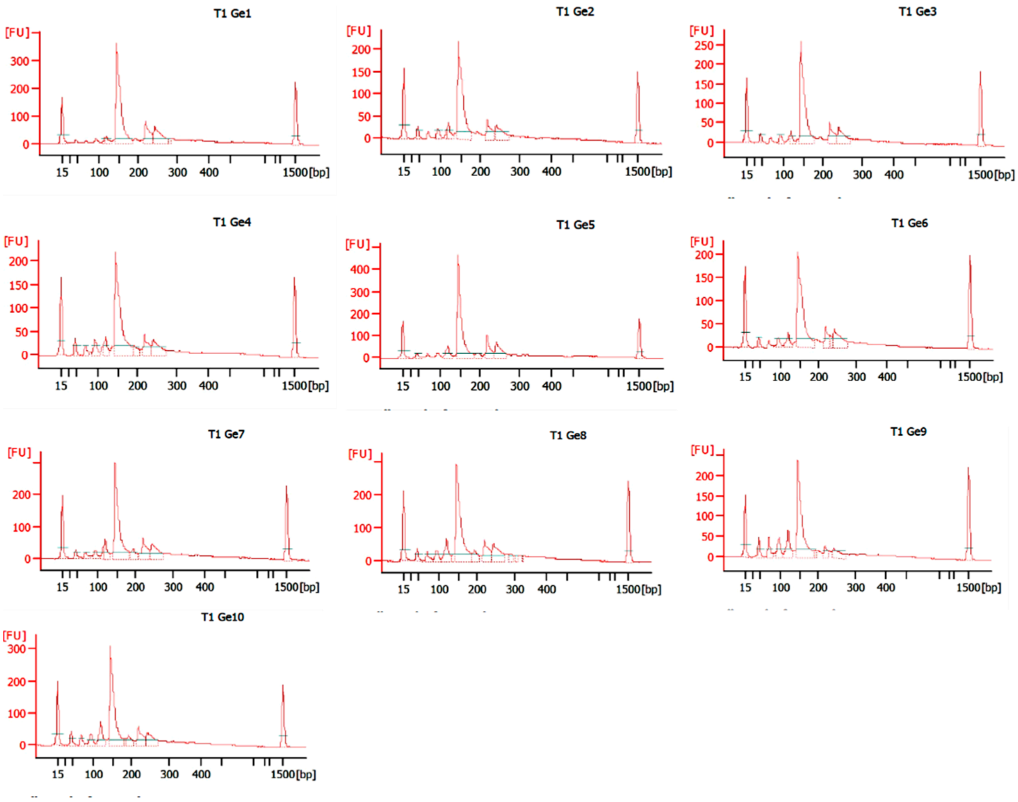


**Figure S3B.** Automated electrophoresis of Chr1-HSATII PCR amplicons in control samples
